# Neuroplastic effects of a selective serotonin reuptake inhibitor in relearning and retrieval

**DOI:** 10.1016/j.neuroimage.2021.118039

**Published:** 2021-04-20

**Authors:** M.B. Reed, T. Vanicek, R. Seiger, M. Klöbl, B. Spurny, P. Handschuh, V. Ritter, J. Unterholzner, G.M. Godbersen, G. Gryglewski, C. Kraus, D. Winkler, A. Hahn, R. Lanzenberger

**Affiliations:** Department of Psychiatry and Psychotherapy, Medical University of Vienna, Austria

**Keywords:** Neuroplasticity, fMRI, SSRI, Learning, Insula, Angular gyrus

## Abstract

Animal studies using selective serotonin reuptake inhibitors (SSRIs) and learning paradigms have demonstrated that serotonin is important for flexibility in executive functions and learning. SSRIs might facilitate relearning through neuroplastic processes and thus exert their clinical effects in psychiatric diseases where cognitive functioning is affected. However, translation of these mechanisms to humans is missing. In this randomized placebo-controlled trial, we assessed functional brain activation during learning and memory retrieval in healthy volunteers performing associative learning tasks aiming to translate facilitated relearning by SSRIs.

To this extent, seventy-six participants underwent three MRI scanning sessions: (1) at baseline, (2) after three weeks of daily associative learning and subsequent retrieval (face-matching or Chinese character–noun matching) and (3) after three weeks of relearning under escitalopram (10 mg/day) or placebo. Associative learning and retrieval tasks were performed during each functional MRI (fMRI) session. Statistical modeling was done using a repeated-measures ANOVA, to test for content-by-treatment-by-time interaction effects.

During the learning task, a significant substance-by-time interaction was found in the right insula showing a greater deactivation in the SSRI cohort after 21 days of relearning compared to the learning phase. In the retrieval task, there was a significant content-by-time interaction in the left angular gyrus (AG) with an increased activation in face-matching compared to Chinese-character matching for both learning and relearning phases. A further substance-by-time interaction was found in task performance after 21 days of relearning, indicating a greater decrease of performance in the placebo group.

Our findings that escitalopram modulate insula activation demonstrates successful translation of relearning as a mechanism of SSRIs in human. Furthermore, we show that the left AG is an active component of correct memory retrieval, which coincides with previous literature. We extend the function of this region by demonstrating its activation is not only stimulus dependent but also time constrained. Finally, we were able to show that escitalopram aids in relearning, irrespective of content.

## 1 Introduction

One of the many core functions of memory is the ability to use previously acquired information and experiences to enable successful adjustment to environmental changes. Previous studies on learning in animals and humans demonstrated the importance of serotonin (5- hydroxytrypatmin, 5-HT) and neuroplasticity in memory, learning and relearning (Thorndike 1931; Tolman 1932; Skinner 1938; Rose 1969; Kandel 1981; Robertson 1982; Shanks et al., 1994; Kandel 2001; Sampaio-Baptista et al. 2017). In recent years these mechanisms involved in learning have been further analyzed with the help of magnetic resonance imaging (MRI). Changes in the structural parameters of gray matter (GM) and white matter (WM) were found in a myriad of different learning tasks, spanning over diverse learning periods. It is further shown that GM and WM changes are susceptible to experience- dependent structural plasticity throughout life (Taubert et al., 2011; Valkanova et al., 2014). Even after a short 2 h period of learning, diffusion tensor imaging was able to detect microstructural changes after completing a spatial learning task. Furthermore, evidence suggests that activity-dependent regulation of myelin may play a role in learning in both animals and humans (Sagi et al., 2012; Sampaio-Baptista et al. 2017). Psychiatric (Strauss et al., 2015; Barch et al., 2016; Morris et al., 2018) and neurological (Swainson et al., 2000; Peters et al., 2009; Grober et al., 2019; Levy-Gigi et al., 2019) conditions are just some of the many factors that can influence ones neuroplasticity and the ability to learn and adapt to an ever-changing environment (Nissen et al., 2010; Ehlers 2012).

Serotonin (5-HT) is a monoamine neurotransmitter with a complex and multifaceted biological function, which has been implicated in regulation of appetite, sexual function, mood, cognition, reward and executive functions (Young 2007; Cools et al., 2008; Faulkner et al. 2014; Stiedl et al., 2015). DNA methylation of the serotonin transporter gene has also been associated with emotional processing (Frodl et al., 2015). Additionally, selective serotonin reuptake inhibitors (SSRIs) are used as the first line of treatment in psychiatric disorders, increasing 5-HT in the synaptic cleft (Taylor et al., 2018).

Animal studies have shown that 5-HT manipulation affects both up- and downregulation of serotonergic transmission during attentional processes e.g. discrimination tasks or spatial learning (Carli et al. 2000; Moreau et al., 2013). Upon targeted 5-HT depletion in the amygdala of non-human primates, increased sensitivity to aversive feedback in learning and reversal tasks was demonstrated (Rygula et al., 2015). This is similar to the effects of a single dose of citalopram on probabilistic learning in healthy subjects (Chamberlain et al., 2006). Additionally, Nord et al. (2013) have shown that despite the variation in doses applied, SSRI research in animals and humans are largely in agreement.

In healthy humans, after tryptophan depletion (TD), 5-HT’s precursor amino acid, memory consolidation is reduced (Riedel et al., 1999). Likewise, after TD remitted depressive patients exhibit a relapse (Neumeister et al., 2004) as well as impaired learning and memory (Hayward et al., 2005; Merens et al., 2008). Moreover, it results in dampened visual discrimination during learning and reverse learning (Rogers et al., 2000). These effects are hypothesized to arise from the inhibitory effects on immediate increases of 5-HT transmission (Blier et al., 1990).

Numerous studies have examined the acute effects SSRIs have on cognitive function e.g. (Kuo et al., 2016; Levy et al., 2019). For instance, one study found that after a single dose of escitalopram, SSRIs impaired the cognitive flexibility in set-shift task whereas in contrast, an enhanced response to a stop cue was also discovered (Skandali et al., 2018). Harmer et al. (2003) demonstrated that after a single infusion of citalopram healthy subjects detected a higher number of facial expressions of fear and happiness compared to placebo. A single dose of citalopram has further been shown to increase the attention to positive, socially relevant stimuli in a visual probe task (Browning et al., 2007), whereas, in a 3-week SSRI intervention, healthy subjects taking SSRIs were able to significantly lower their threshold for visual perception in a visual attention paradigm (Lansner et al., 2019). Despite the vast amount of studies investigating the acute effects SSRIs have on neuroplasticity and cognitive processes, the investigation of prolonged assessment effects has been scarce. Graf et al. (2016) revealed unaffected or enhanced neural prediction error processing in healthy male participants after a 7-day paroxetine intake.

Even though SSRIs are administered therapeutically for a prolonged period, little is known about the long-term neuroplastic effects SSRIs have on emotional learning and reversal learning in humans. To resolve the lack of knowledge about prolonged effects of SSRIs on cognition and neuroplasticity in humans we aimed to detect effects of SSRIs in a novel association learning paradigm either with emotional valence (faces) or symbol-word matching (Chinese character to German noun). In combination with testing functional activity of the underlying neuronal networks, the paradigm enabled us to investigate neuronal activation during learning and retrieval indirectly, via the blood-oxygenation- level dependent (BOLD) response (Hillman 2014). Further, the longitudinal framework of the study allowed for the investigation of prolonged effects SSRIs may have on neuroplasticity. We hypothesized that escitalopram compared to placebo would significantly modulate emotionally valenced relearning.

## 2 Methods

One-hundred thirty-eight healthy right-handed subjects (73 female, mean age ± SD: 25±6 years) participated in this study (see results for final sample). Participants’ general health was assessed based on medical history, physical examination and a structured clinical interview from DSM-IV (SCID). Exclusion criteria included any medical, psychiatric or neurological illness, current or former substance abuse, smoking, first degree relatives with a history of psychiatric illness, color blindness, non-European ancestry, MRI contraindications and knowledge of Kanji or Hanzi (Japanese or Chinese characters). Moreover, subjects were also excluded for not being able to comply with the study-imposed schedule, reported study medication side effects, technical issues and structural anomalies or upon their own request at any time during their participation. To minimize the induction of neuroplasticity due to undesired factors, participants were told not to travel during participation. Furthermore, participants were instructed that each home-learning session should be undergone at the same time of day and to make sure that they were adequately rested, i.e., not to start a learning-session right before going to sleep, right after waking up or after an extensive work load. Each participant’s home-learning session was logged and reviewed before each MRI session, failure to complete each of the 21 sessions led to exclusion from the study.

This study was approved by the ethics committee of the Medical University of Vienna (EK Nr.: 1739/2016) and was performed in accordance with the Declaration of Helsinki (1964). The study was registered at clinicaltrials.gov with the identifier NCT02753738. All participants gave written consent to partake in this study, were insured and received reimbursement for their participation. The distributions of sex and participation in between groups were tested using Fisher’s exact and that of age with a Kruskal–Wallis test.

### 2.1 Study design

**Fig. 1:**
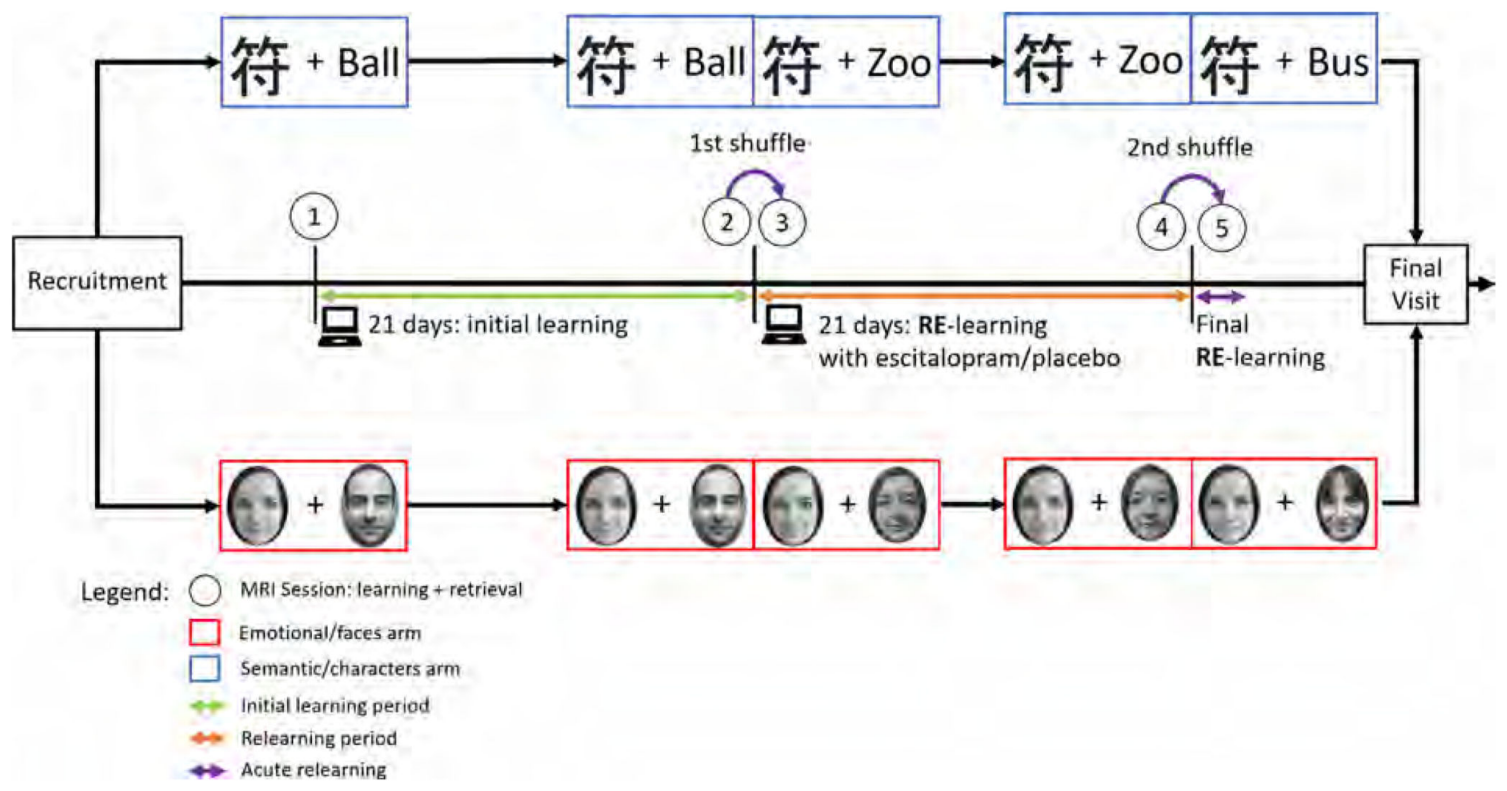
Illustration of the study design: the study comprised of 3 MR sessions, each session contained a learning and relearning scan. The 2nd and 3rd MR sessions contained 2 MR learning and retrieval scans. Before the 1st MRI appointment, each participant was randomly assigned to one of 2 groups (faces/Chinese characters). Each subject then completed their first onsite learning and retrieval session in the MRI scanner (1st MR session). Thereafter, they were instructed to learn further associations at home for 21 days and returned for a 2nd MRI appointment during which they underwent another learning and retrieval session (2nd MR session) before the pairs were shuffled and the relearning phase was initiated in the scanner (3rd MR session). Another 21 days of relearning at home took place during which participants received double-blind treatment with escitalopram 10 mg daily or placebo. Subjects then returned for the 3rd MRI session which again included a relearning and retrieval paradigm (4th MR Scan). To conclude the last MRI session, pairs were shuffled again, and participants underwent a second relearning paradigm under medication or placebo (5th MR scan). Subjects successfully completed the study with a final visit where general health was assessed once again.

Subjects were recruited for a randomized, double-blind, placebo- controlled longitudinal study. See Fig. 1 for a graphical overview of the study design. Each subject was randomly assigned to one of four study arms. These differed with regard to learning content in the associative learning task (Chinese character matching or face matching) and double-blind treatment during the relearning phase (SSRI or placebo). The learning groups had to learn pairwise associations of either Chinese characters to random German nouns or between two faces (see below). During the study three MRI sessions each separated by 21 days were conducted. Learning and retrieval tasks were carried out at the baseline MRI session, for 21 consecutive days at home via online platform and during the 2nd MRI examination. The day after the 2nd MRI, half of the study participants (randomized classification within each learning group) received escitalopram (Cipralex® Lundbeck A/S) 10 mg orally per day for the final 21 days of the study. This enabled us to specifically assess the effects that SSRIs exert on relearning processes. The SSRI escitalopram is one of the most prescribed treatments for numerous brain disorders such as depression and anxiety. It has been shown that escitalopram facilitate multiple effects on learning and memory (Harmer et al., 2002). However, this is most likely common to all SSRIs (Seo et al., 2014). The other half of the participants received identical placebo tablets for 21 days as a control group (double-blind, both substances provided by the pharmacy of the Medical University of Vienna). Venous blood was drawn from the cubital vein to measure citalopram plasma levels at 3 time points: 7 days, 14 days and 21 days after start of therapy (i.e., the before the 3rd MRI). Citalopram plasma levels were assessed with liquid chromatography-tandem mass spectrometry (LC-MS/MS) at the Clinical Department of Laboratory Medicine of the Medical University of Vienna. Furthermore, participants were given the option to take a short break outside of the scanner between both 2nd and 3rd and the 4th and 5th MRI sessions to combat fatigue from long scan times under task engagement.

### 2.2 Associative learning paradigm

**Fig. 2:**
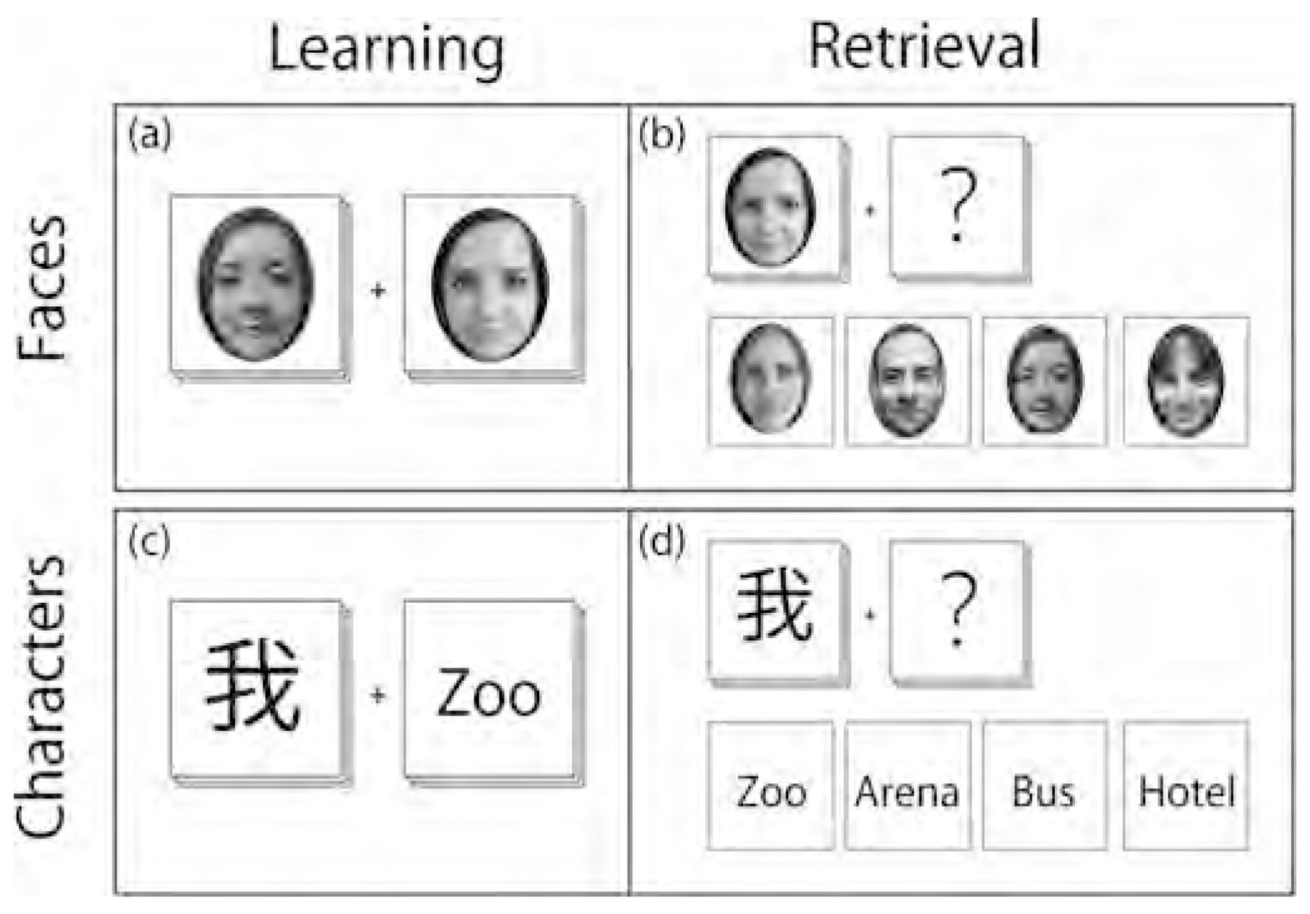
Illustration of the associative learning paradigm: (a) and (c) show the subjects views of the learning paradigm in the scanner for both groups respectively, whereas (b) and (d) represent the retrieval task for both groups. For both the learning and retrieval task, subjects had 8 s to learn or choose their subjectively correct association.

Throughout the study, each participant was required to perform an associative-learning task, learning either emotional (facial) or cognitive (character) content of the pairs presented, which aimed to activate neuronal circuits mediating cognition and emotions. The emotional/faces group pairs displayed, indicated positive and neutral valence. Throughout the study, participants were required to learn and relearn 200 separate image-pairs per group, see Fig. 2a and c. Each learning session, either at home or in the scanner, consisted of a subset of 52 image pairs (pseudorandomized with replacement). Each pair was presented in 13 blocks of 4 image pairs lasting 8 s each. For each MRI session, between each learning sub-block, a control block was also presented, where the images above were distorted heavily. For this task participants were not required to elicit a response to the stimulus presented.

### 2.3 Associative retrieval paradigm

Immediately after each learning session in the scanner, participants were required to complete a retrieval task to quantify the extent of content learned. In accordance with the learning task, each subject was presented one half of a random pair selected from a subset of all previously learned associations. Correct associations should be selected from a given set of 4 possible answers within an 8 s period (see Fig. 2b and c). The control condition comprised of correctly matching heavily distorted images. This was repeated 52 times in the same design as the learning paradigm. All faces were derived from the “10k Adult Faces Database” (Bainbridge et al., 2013). The Characters were selected at random and were not direct translations to the words presented. For this paradigm, subjects were required to respond to the stimulus shown. This was done by using a MR-compatible keyboard with 4 keys. Each key represented one of the possibilities presented.

### 2.3 MRI acquisition and processing

All neuroimaging data was recorded using a Siemens Prisma 3T scanner (Siemens, Erlangen, Germany) equipped with a 64- channel head coil. During each MRI session, the learning, relearning and retrieval tasks were performed using the following parameters: TE/TR = 30/2050 ms, GRAPPA 2, 210 × 210 mm field of view, 100 × 100 pixel in-plane resolution, 35 axial slices of 2.8 mm (25% gap) which was oriented parallel to the anterior-posterior commissure line. The data was preprocessed using SPM12 (The Wellcome Centre for Human Neuroimaging, www.fil.ion.ucl.ac.uk), BrainWavelet toolbox (Patel et al., 2014) and custom scripts in MATLAB R2018b. Slice- timing correction was performed to the temporally middle slice, following a two-pass realignment. Images were normalized to the standard Montreal Neurological Institute (MNI) space and a custom binary mask was applied to exclude all non-brain voxels. The BrainWavelet toolbox was used to correct for nonlinear artifacts using the “chain search” parameter set to “harsh” and a “threshold” of “20” (Patel et al., 2014) was set to adjust for the application to unsmoothed data and a decreased signal-to-noise ratio stemming from GRAPPA acceleration. The images were then gray-matter-masked and smoothed with a Gaussian kernel of 8 mm full-width at half-maximum. Six movement, WM and cerebrospinal fluid (CSF) parameters were used as regressors for the 1st-level GLM. The WM and CSF parameters were calculated using a principle component analysis, where the first 5 components were taken as regressors. Furthermore, the autocorrelation was set to “FAST” (Olszowy et al., 2019).

### 2.4 Statistical analysis

Statistical analysis of the fMRI and in-scanner performance data was performed using SPM12 and MATLAB R2018b respectively. First, we aimed to indirectly elucidate task-specific changes in BOLD-derived neuronal activation for learning and retrieval for each subject and session. For the learning task, the contrast learning versus control was carried to the group level with the conditions modeled as a block design. As for the retrieval task the contrasts correctly learned versus control were set in an event related model. On the group level we tested for main and interaction effects using a 2 × 2 × 5 (substance × content × time) repeated measures ANOVA (rmANOVA) for both learning and retrieval tasks. “Substance” consists of verum/placebo, “content” of faces/Chinese characters and “time” comprises of learning and relearning phases of the study (t1, baseline; t2, final learning phase; t3, start of the 1st relearning phase; t4, final relearning phase; t5, final relearning phase. Second- level analyses were corrected for multiple testing using Gaussian random field theory as implemented in SPM12 and the threshold for significance was set at *P*⩽0.025 family-wise error (FWE)-corrected according to Chen et al. (2019) at the cluster-level following *P*⩽0.001 uncorrected at the voxel-level. To analyze the task performance from the in-scanner retrieval task a rmANOVA was conducted to test for substance × content × time and lower-level interactions in the same manner as in used to analyze the fMRI data using the percent of correctly retrieved pairs. All post-hoc comparisons for both tasks and the in-scanner performance were adjusted for multiplicity using the Bonferroni correction for multiple conditions.

## 3 Results

### 3.1 Study population

Eighty-four subjects successfully completed all learning phases. From those, 4 further subjects were dropped due to data quality issues, 3 were dropped as their escitalopram plasma levels were under the measurable threshold and 1 final subject was dropped due to a missing log file. The remaining 76 subjects used in the analysis were 25.3 ± 4.7 (mean ± SD) years old and comprised of 45 women. There were no significant group differences regarding age, education, sex or participation numbers (*P* > 0.3). See Table 1 for detailed demographics and participant stratification.

63% of the participants had completed their A-levels, 31.1% had already completed at least one university degree, 2.3% completed their apprenticeship, another 2.3% completed the mandatory education and 1.3% had not finished any level of education.

### 3.2 The effects of substance-related associative learning

**Fig. 3:**
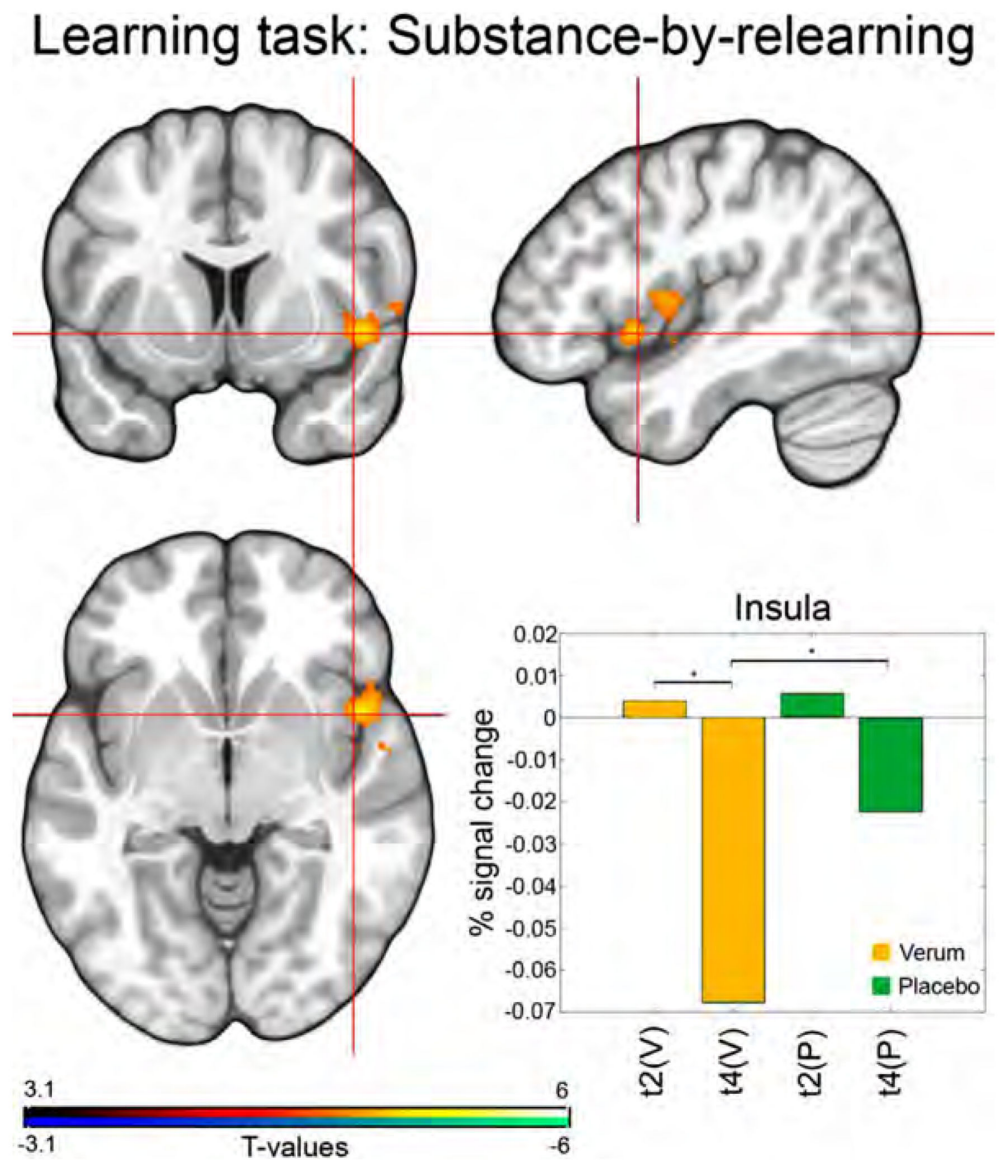
Substance-by-time interaction effect on the learning task. After 21 days of relearning under a daily dose of 10 mg escitalopram, the verum group (orange bars) showed a significantly greater deactivation (cluster + Bonferroni corrected *P* < 0.025) of the right insula (T = 4.36; *p* = 0.02) in comparison to placebo (green bars). For each cohort, the median parameter estimates for the contrast (stimuli > control) are plotted (percent signal change as calculated in Luo et al., 2003). The asterisk indicates significant differences. Abbreviations: P, Placebo; V, Verum; t, time; t2, learning phase scan; t4, relearning phase scan.

For learning and retrieval no significant 3-way interaction effects between sessions, substance and learning content were found. However, while assessing the learning paradigm a significant 2-way interaction (time-by-substance) was found in the right insula, independent of learning content. This showed a significantly greater deactivation in the verum group when contrasted with the placebo group after relearning for 21 days when compared to after learning for 21 days T(t2 - t4) = 4.36; *p* = 0.02 (Fig. 3). In contrast, no acute relearning substance- by-time interaction effects were detected (t2 vs t3 or t4 vs t5). Moreover, no content-by-time interaction effects were found for the learning task and no significant differences between learning performance and substance or content were discovered.

### 3.3 The effects of content-related associative learning on retrieval

**Fig. 4:**
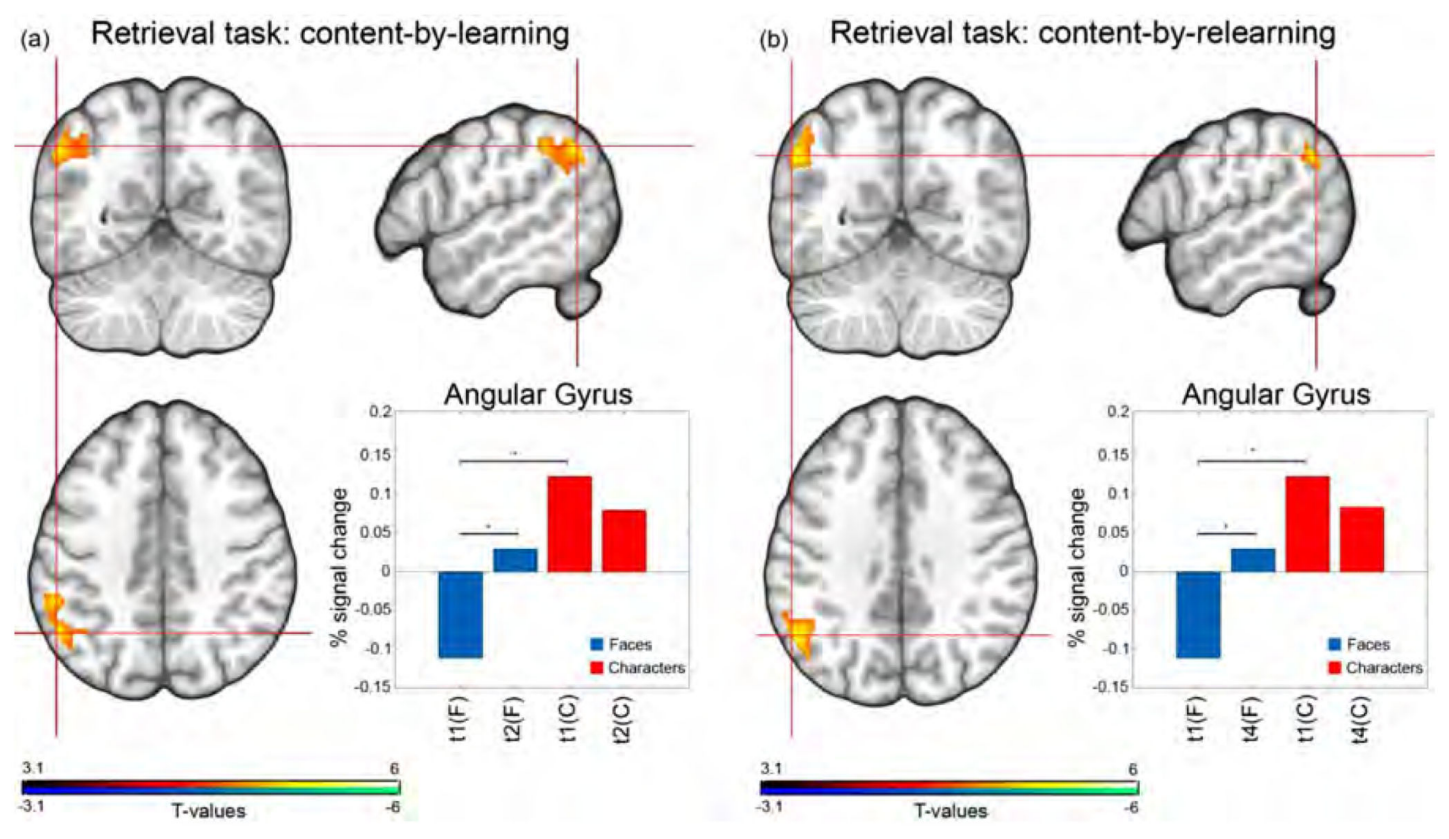
Content-by-time retrieval interaction effects were discovered for both the learning and relearning phases when contrasting correctly retrieved pairs > control pairs in the left AG. After 21 days of learning (a) and again 21 days of relearning (b) the face matching group (blue bars) showed an increase in task activation over time while the character group (red bars) showed a decrease in task activation over time (cluster + Bonferroni corrected *P* < 0.025; learning: *T* = 4.44; *p* = 0.001 and relearning: *T* = 4.85; *p* = 0.01.). For each group, the parameter median estimates for the contrast (correctly learned - control) are plotted. The asterisk indicates significant differences. Abbreviations: C, characters; F, Faces, t, time; t1, baseline; t2, learning phase; t4, relearning phase.

For the retrieval task, a content-by-time interaction effect was discovered in the left AG for both learning T(t1 - t2) = 4.44; *p* = 0.001 and relearning phases T(t1 - t4) = 4.85; *p* = 0.01 of the study across both substances. This interaction showed an increase of activation for the faces group whereas a decrease in activation was found in the character group. Thus, the pronoucned difference in activation between the retrieval of faces and characters at baseline was largely decreased after 21 days of memory consolidation and lasted throughout the subsequent 21 days of relearning including escitalopram administration, see Fig. 4, bar chart. No other significant retrieval interaction effects were discovered.

### 3.4 The effects of substance-related effects on task performance

**Fig. 5:**
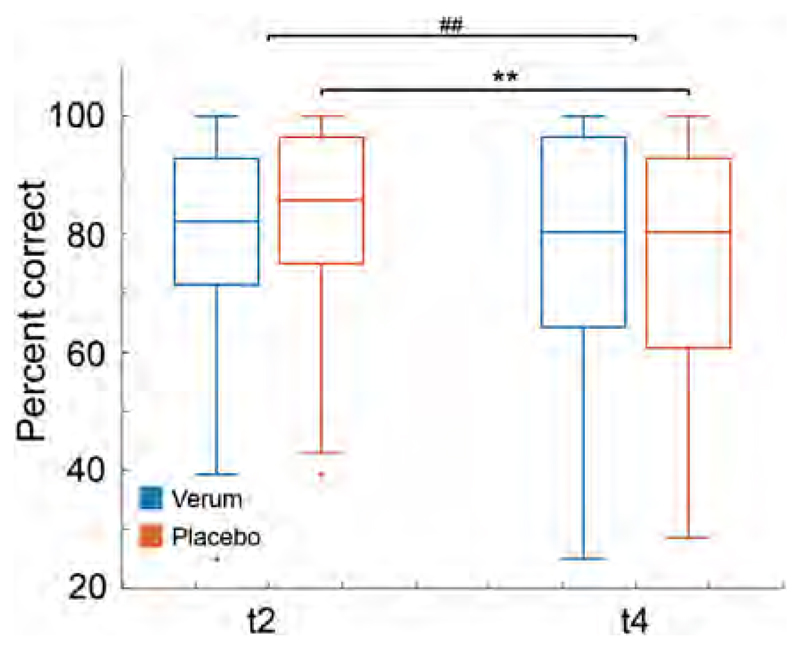
A substance-by-time interaction (*F*_4, 72_ = 3.43; *p* = 0.0093) was found between 21 days after learning (t2) and 21 days after relearning (t4) while receiving either 10 mg/day escitalopram or placebo. The post-hoc tests indicate a greater decrease in learning performance in the placebo group, irrespective of content when compared to the verum group *T* = 2.85; Bonferroni corrected *p* = 0.0476. The asterisk indicates significant post-hoc differences, whereas the hash indicates a significant interaction.

No 3-way interaction was found for the in-scanner task performance. However, a 2-way (substance-by-time) interaction was observed between t2 (21 days of learning) and t4 (21 days of relearning) while receiving either placebo or 10 mg/day escitalopram, *F*_4, 72_ = 3.43; *p* = 0.0093. Post-hoc tests revealed a greater decrease in the retrieval task performance for the placebo group ( *T* = 2.85; *p*(Bonferroni) = 0.0476) in comparison to the verum group across both task contents (faces and character-word pairs). See Fig. 5. No other interaction effects where found.

Finally, participants subjectively rated both learning and relearning phases, were 52.0% stated that the initial phase of learning was easier, whereas 37.7% found the subsequent phase of relearning easier. 10.4% of participants withheld their judgment.

## 4 Discussion

This study aimed to investigate neuroplastic effects of SSRIs on learning, relearning and memory retrieval as assessed with fMRI. Specifically, for the learning task, a substance-by-time interaction effect was discovered in the right insula. Furthermore, a group-by-time interaction effect was found in the left AG while performing the retrieval task. These results suggest that relearning is modulated by the SSRI escitalopram and memory retrieval seems to be dependent on the stimulus of the task and the consolidation of memory. Furthermore, relearning performance may be influenced by escitalopram.

The insula plays an important role in explicit motivation (Balleine et al. 2000; Berridge et al. 2003) or the desire to engage in a certain behavior, working memory (Göbel et al., 2019) and affective processing (Teckentrup et al., 2019). Additionally, the insula, together with hippocampal, parahippocampal and thalamic regions are all robustly activated by tasks requiring explicit processing (Scheuerecker et al., 2007; Deeley et al., 2008; Knight et al., 2009; Etkin et al. 2011). Furthermore, dysfunctions of the insula were demonstrated in psychiatric diseases exhibiting deficits of explicit motivation such as major depressive disorder (Takahashi et al., 2010; Stratmann et al., 2014), bipolar disorder (Najt et al., 2016; Wise et al., 2017) and schizophrenia (Wylie et al. 2010).

After SSRI treatment depressed patients exhibited a lower activation in the right insula (Wang et al., 2012) in comparison to healthy controls (HCs) when viewing positive pictures. This effect was also found in HCs, as Simmons et al. (2009) found a greater deactivation in the insular cortex during anticipation of valenced images after SSRI administration. This is in line with the findings of our learning task, where we observed a greater decrease in the task-induced BOLD response in the escitalopram group compared to placebo. In contrast, Arce et al. (2008) found a somewhat unexpected effect of a greater insula activation in HCs in the escitalopram group, where they argue that only the emotion of anger was contrasted and therefore not valence dependent. Anderson et al. (2011) also reported a similar result in HC after an intravenous citalopram pretreatment, whereby the insula also showed an increased activation to sad faces. Chiarotti et al. (2017) hypothesized that SSRIs amplify the influence of a condition on mood, which can be related to our finding demonstrating greater deactivation of the insula after prolonged intake of escitalopram. Hence, SSRIs dependent neuroplasticity might facilitate greater reactivity of the insula. Emotional regulation and processing is disrupted in depressed patients (Ebneabbasi et al., 2020). Moreover, depression is associated with negative evaluation and assessment of emotional life (Harmer et al. 2013). Harmer and Cowen argue that such a psychological process might involve relearning a range of emotional associations and this process would require a certain amount of time and exposure. Therefore, our finding of increased insular reactivity during learning in healthy volunteers could contribute to improvements of mood over time in patients with depression – as seen under antidepressant therapy with SS- RIs. In terms of mechanisms of actions of SSRIs, our findings emphasize the importance of learning and serotonergic modulation of the insula (Nagai et al., 2007).

Our second finding demonstrates that the AG plays a central role in memory retrieval (Seghier 2013) and successful recollection of memories (Ciaramelli et al., 2008). The left AG engages in all aspects of semantic processing, which requires concept retrieval (Binder et al., 2009) and language comprehension (Price 2010; Seghier et al., 2010). Furthermore, the AG monitors retrieval output, whereby task-based activation increases when the subject is confident that the item was recognized (Cabeza 2008; Ciaramelli et al., 2008). Boninici et al. found that when selectively inhibiting the left AG the retrieval of autobiographical memories was disrupted and subjects could recall fewer details. Taken together, previous evidence shows that the AG plays a central role in memory retrieval independent of the task’s valence. Here, we extend the available knowledge by demonstrating that the AG also encodes a time-dependent aspect of memory retrieval. Specifically, the pronounced BOLD activation of the AG between faces (Joassin et al., 2011) and characters (Price et al. 2011) at baseline is substantially reduced when memory has been consolidated for a longer time period (in our case 21 days). This could indicate that as the memories become more solidified, the AG becomes either less involved in the retrieval process or the retrieval of these memories becomes less arduous.

Previous studies suggest that the SSRI escitalopram facilitates relearning and memory consolidation in HC (Harmer et al., 2002; Brown et al., 2012). Further, studies conclusively suggest enhancement of neuronal plasticity as a result of treatment with SSRIs in both animal models (Vetencourt et al., 2008, 2011, Piubelli et al., 2011) and humans (Nitsche et al., 2009). Following 21 days of relearning and a daily intake of escitalopram 10 mg, our in-scanner performance showed improved accurate retrieval of image pairs. Alternatively, the placebo group had a significantly greater decrease in retrieval performance. The BOLD response in both insula and angular gyrus did not correlate with the learning performance. This may indicate that there is no direct relationship between retrieval performance and BOLD response in these particular brain regions.

Unlike alphabetical words Chinese characters are much more similar to faces, which exhibit a nonlinear square configuration, and hence contain more configural information (Yeh et al. 2002). In this study the face images were also converted to greyscale which make the face images even more similar to Chinese characters in terms of color, intensity and luminance. These factors provide a highly specific contrast but may contribute to an overlap in task-related group activations, as they share many regions of interest when compared to one another. Furthermore, similar regions in the brain are active when processing characters (Ding et al., 2003; Peng et al., 2004; Liu et al., 2008) and faces (Kanwisher et al., 1997; Gauthier et al., 2000). This might further contribute to the lack of content differences in our results.

As serotonin possesses vasoconstrictor properties, SSRIs could theoretically lead to alterations in the BOLD signal independent of neuronal effects. However, numerous studies show that changes in cerebral blood flow modulated by SSRIs and fenfluramine (Meyer et al., 1996; Holschneider et al., 2000) seem to be driven by changes in local field potentials (Eimon et al., 2018). The sample size of 76 participants, although large at first glance, should be discussed as a limitation. When taking the study design into account, the size of each subgroup is similar to common fMRI investigation that use pharmacological interventions, see Table 1. This was a result of randomization and dropouts. Hence, it is possible that more subtle effects were missed in this work. Even though compliance of study drug intake was controlled via assessment of citalopram plasma levels and participants were only included in the final analysis if the medication was verified at the 3rd MRI session. This however does not exclude the possibilities of irregularities in self-administration. Another possible cofounding effect could be due to the back-to-back learning/retrieval and relearning which could result in fatigue or learning saturation. This was partly compensated for by offering a short break out of the scanner between learning and relearning sessions. Furthermore, the 21 day SSRI intake must also be discussed as a limitation as depressive patients are administered SSRI for much longer periods. It usually takes 2-3 weeks of SSRI intake for clinical and neuronal changes to become apparent (Godlewska et al., 2016). Nevertheless, Arce et al. (2008) applied a 10 mg/day dose of escitalopram over 21 days in a cross-over fashion and was able to assess escitalopram induced changes in the insula and amygdala using an emotional discrimination paradigm. Therefore, 21 days or 3 weeks of daily escitalopram intake was deemed an adequate timeframe to assess functional and structural changes.

## 5 Conclusion

In this study we observed that escitalopram modulated the BOLD activation (a proxy for the neuronal response) of the insula during relearning, with a stronger deactivation as compared to placebo. In addition, we demonstrated that BOLD-derived neuronal activation of the angular gyrus between retrieval of emotional and cognitive information varies as a function of memory retrieval. Furthermore, considering that the insula plays an important role in psychiatric disorders, our paradigm and findings provide an interesting framework to assess SSRI-dependent effects in patients. Our findings contribute to a better understanding of how SSRIs affect learning and relearning, which might be important therapeutic mechanisms of action in patients with psychiatric diseases.

## Figures and Tables

**Table 1:** Detailed demographics for all participants included in the final analysis. No age, gender, sex or participation between groups were found.

	Faces		Characters	
***N***	39		37	
**Age in years [mean + SD]**	25.6 ± 5.3		25.4 ± 4.5	
**Gender [M/F]**	14/25		17/20	
Education [0,1,2,3,4] *	0,2,1,17,19		1,1,0,26,9	

	**Verum**	**Placebo**	**Verum**	**Placebo**

***N***	16	23	18	19
**Age in years [mean ± SD]**	25.5 ± 5.2	25.7 ± 5.5	25.3 ± 5	25.5 ± 4.1
**Gender [M/F]**	7/9	7/16	7/11	10/9
Education [0,1,2,3,4] *	0,1,1,7,7	0,1,0,10,12	0,0,0,14,4	1,1,0,12,5

* 0 = No completed education, 1 = Completed mandatory education, 3= Completed high school, 4 = Completed university degree.

## Data Availability

Due to data protection laws processed data is available from the authors upon reasonable request. Please contact rupert.lanzenberger@meduniwien.ac.at with any questions or requests.
